# The role of organoids in cancer research

**DOI:** 10.1186/s40164-023-00433-y

**Published:** 2023-08-03

**Authors:** Zhen Fang, Peijuan Li, Fengying Du, Liang Shang, Leping Li

**Affiliations:** 1 Department of Gastroenterological Surgery, Shandong Provincial Hospital of Shandong First Medical University, Jingwuweiqi street, 324, Jinan, 250021 Shandong China; 2 Department of Digestive Tumour Translational Medicine, Engineering Laboratory of Shandong Province, Shandong Provincial Hospital, Jinan, 250021 Shandong China; 3 Medical Science and Technology Innovation Center, Shandong First Medical University, Shandong Academy of Medical Sciences, Jinan, 250021 Shandong China; 4 Emergency Department, The First Affiliated Hospital of Dalian Medical University, Dalian, Liaoning China

**Keywords:** Organoid, Cancer, Precision Medicine, Drug screening, Organoid Biobank

## Abstract

Organoids are established through in vitro 3D culture, and they can mimic the structure and physiological functions of organs or tissues in vivo. Organoids have attracted much attention in recent years. They can provide a reliable technology platform for cancer research and treatment and are a valuable preclinical model for academic research and personalized medicine. A number of studies have confirmed that organoids have great application prospects in new drug development, drug screening, tumour mechanism research, and precision medicine. In this review, we mainly focus on recent advances in the application of organoids in cancer research. We also discussed the opportunities and challenges facing organoids, hoping to indicate directions for the development of organoids in the future.

## Background

Organoids are a model based on a three-dimensional (3D) cell culture system in vitro that is highly similar to source tissues or organs in vivo [1–5]. Organoids were named the 2017 Technology of the Year in Life Sciences by the journal *Nature Methods*. Although organoids are not real human organs, they can simulate real organs in terms of structure and function and can simulate the interactions and spatial position relationships between cells and between cells and their surrounding matrix [6, 7].

Organoids are widely used in cancer research [3, 6, 8–14]. The emergence of organoid models has made it possible to culture certain cancerous tissues in vitro. Compared with the traditional 2D cell culture model, 3D cultured organoids contain a variety of cell types, can form functional “micro-organs”, and can be better used to simulate the development process and physiological and pathological state of organ tissues. Organoids preserve the genetic, phenotypic, and behavioural characteristics of the tumour tissue of origin and have become the most relevant models for drug discovery, tracking treatment response, and personalized medicine [15–23]. It has broad application prospects in basic cancer research and clinical diagnosis and treatment.

Organoids are currently divided into two categories: tissue-derived organoids and pluripotent stem cell-derived organoids [6, 17, 19, 24]. Patient-derived tumour organoids (PDTOs) are commonly used in disease modelling, research and drug screening of tumour patients. Tumour organoids can be obtained by culturing biopsied, aspirated, or surgically resected tissue in Matrigel for several weeks. Tumour organoids maintain the heterogeneity of the source tumours and patients; moreover, the shape and size of organoids are basically uniform among individuals. Thus, they provide a good technical platform for research on tumour pathogenesis, drug screening, precision medicine and other fields [25–28]. In clinical work, before treating cancer patients, organoids derived from tumour tissues can be used to screen sensitive drugs and provide individualized treatment options for cancer treatment.

In this review, we mainly focused on recent advances in the application of organoids in cancer research, especially in precision cancer therapy. We also discussed the opportunities and challenges facing organoids, hoping to point out the direction for future organoid development.

## History of organoids

The earliest events related to organoid development date back to 1907. Professor H. V. Wilson discovered that mechanically separated sponge cells can reassemble and self-organize into new sponge organisms with normal functions. This research demonstrated that adult organisms can successfully develop into new organisms without external assistance and without starting from a specific anatomical stage [29]. This could be considered the source of the development of organoid technology. In 1960, Professor Taylor, A. C. conducted isolation-regeneration experiments to generate different types of organs from isolated amphibian pro-nephros and chicken embryos [30]. In 1961, Verney, E. L. observed the differentiation of embryoid bodies in vitro [31]. In 1981, scientists first isolated pluripotent stem cells (PSCs) from mouse embryos. Stem cell research has grown rapidly since then [32, 33]. In 1987, scientists began to improve cell culture conditions by simulating the microenvironment in vivo. Li et al. demonstrated that mammary epithelial cells formed 3D ducts and lumens when cultured in extracellular matrix (ECM) extracts of Engelbreth-Holm-Swarm (EHS) mouse sarcoma [34]. In the same year, a study found that alveolar type II epithelial cells were able to maintain their differentiation capacity in the presence of ECM stroma, highlighting the importance of cell-matrix interactions in tissue maintenance and differentiation [35]. In 1987, A. J. Friedenstein discovered mesenchymal stem cells (MSCs). In 1998, scientists isolated and cultured embryonic stem cells from human blastocysts for the first time [36]. From 2006 to 2007, Thomson et al. successfully prepared human induced pluripotent stem cells (induced pluripotent stem cells, iPSCs), which is of great significance to stem cell and organoid research [37–39]. Today, most types of nontumor-derived human organoids can be developed from embryonic stem cells (ESCs), MSCs or iPSCs [40]. The rapid progress of stem cell research has brought new vitality to organoid research.

The milestone moment was in 2009, when the Hans Clevers team in the Netherlands used a single leucine-rich repeat-containing receptor 5+ (LGR5+) intestinal stem cell to culture cells in vitro to establish an intestinal organoid with an intestinal crypt-villus structure, which opened a new era of organoid technology development [41]. As early as 2007, Hans Clevers discovered that the stem cells of the small intestine and colon were LGR5 + cells [42]. In 2009, the experimental team cultured crypt cells isolated from mouse intestines in a 3D Matrigel culture system containing ENR (EGF, Noggin, R-spondin). They found that under the cultivation of this culture system, crypt cells can form microstructures similar to the crypt-villi-like complexes of the intestines. Using this culture system to culture a single LGR5 + intestinal stem cell isolated and identified earlier, it was found that organoids with the abovementioned special structure can also be formed, and the organoid still has LGR5 + intestinal stem cells [41]. Therefore, the model could well simulate the morphology, structure and function of the small intestine in vivo. The successful establishment of the small intestine organoid system has opened a new chapter in organoid research.

Since 2009, organoids of the stomach, retina, brain, liver, kidney, pancreas, breast, and fallopian tubes have been successfully grown [43–47]. In 2011, Hans Clevers established tumour organoids using organoid technology. Based on the culture conditions of mouse colonic crypts, they cultivated organoids of the human small intestine and colon by adjusting the culture environment. After further optimization and adjustment, tumour organoid models were established from colon adenoma and adenocarcinoma and Barrett’s oesophagus [48]. In 2014, a study was the first to successfully grow six prostate cancer organoids using tumour samples from patients with metastatic prostate cancer. Tumour samples were largely consistent with the genetic signature of the corresponding organoids. In addition, organoids were successfully grown from a patient’s circulating tumour cells [10]. In 2015, Hans Clevers’ team used CRISPR/Cas9 technology to create colorectal cancer (CRC) organoids. In the same year, he also reported tumour organoids established from 20 CRC patient samples. High-throughput sequencing analysis found that the gene change profile of organoids was highly consistent with the results of large-scale mutation analysis of CRC [49]. These organoids could be used for large-scale drug screening to detect some genetic changes associated with drug sensitivity, which is of great significance for individualized precision medicine. In 2015, Tuveson, D.A. successfully developed a new model system for cultivating normal and cancerous pancreatic cells in the laboratory, which could establish organoid models from normal and tumour mice and human pancreatic tissue [50]. The results of this study provide a new platform for the study of the molecular mechanism of pancreatic cancer. In 2017, Meritxell Huch reported the successful cultivation of the first human primary liver cancer organoids [15]. The effect of 29 anticancer drugs, including existing drugs and new drugs in development, was tested using liver cancer organoids, and the preliminary results were encouraging. This research achievement was a milestone in the development history of liver cancer research.

**Fig. 1:**
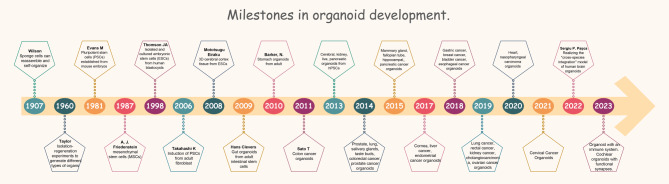
Milestones in organoid development

In 2018, Hans Clevers developed a method for long-term culture and maintenance of breast epithelial organoids, and the success rate of breast cancer organoid culture was over 80%. Their team established more than 100 breast cancer primary tumour and metastatic tumour organoids and used them for analysis of gene expression and drug sensitivity experiments [51]. The study confirmed that breast cancer organoids maintained the histopathological characteristics of the primary tumour well. In 2019, Professor Smith, J.J. established 65 tumour organoids from patients with primary, metastatic or recurrent rectal cancer. The study found that rectal cancer organoids retained the molecular characteristics of their origin tumours, and the results of in vitro drug sensitivity experiments correlated with the clinical responses of tumour patients [6]. Therefore, the findings confirmed that this rectal cancer organoid can be used as an in vitro platform for drug sensitivity experiments. In September 2020, Lee J. et al. successfully produced heart organoids that could beat autonomously [52]. In 2022, Sergiu P. Pașca’s team discovered that transplanting human cortical organoids into rat brains can lead to normal development, realizing the “cross-species integration” model of human brain organoids for the first time (Fig. 1) [53].

## Organoid formation, culture, identification, and storage

**Fig. 2:**
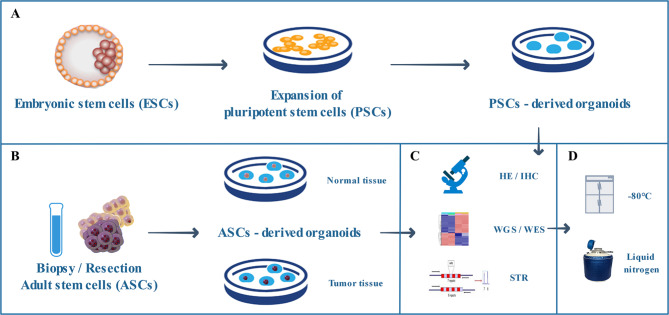
Organoid formation, culture, identification, and storage. **A** PSC-derived organoids. **B** ASC-derived organoids. **C** Identification of organoids. **D** Storage of organoids. PSCs: pluripotent stem cells; ASCs: adult stem cells

According to the source of stem cells, organoids are mainly divided into two types: organoids derived from pluripotent stem cells (PSCs), in other words, either embryonic stem cells (ESCs) or induced pluripotent stem cells (iPSCs), and organoids derived from organ-specific adult stem cells (ASCs), which are tissue-specific resident stem cells (Fig. 2A, B) [54]. Stem cell-derived organoids form through self-organization. They serve as models of organ development, function, and disease and have potential applications in drug development and personalized medicine. However, stem cell self-organization is difficult to control in the absence of external guidance, leading to a general lack of reproducibility in most organoids. Researchers are constantly exploring culture methods that can produce uniform and stable organoid models for better application in clinical research (Fig. 2).

N. Gjorevski et al. used in situ photopatterned hydrogel mechanics and hydrogel microfabrication techniques to control the self-organization process of intestinal stem cells and established intestinal organoids with well-defined shapes, sizes, and cell distributions [55]. In October 2022, a bioengineering device that can be used for long-term organoid culture was reported in the journal *Nature Methods*; this device can evenly diffuse nutrients and oxygen to grow larger, better and more complex organoids [56]. This simple, flexible, and automatable platform made it possible to grow tissue-specific organoids on this platform for extended periods of time.

To obtain human pluripotent stem cell (hPSC)-derived gastrointestinal (GI) organoids, hPSCs need to be de novo differentiated to generate isolated spheroids. In June 2022, a method for simple, reproducible, and scalable small intestinal organoid production based on cryopreserved hPSC-derived mid-hindgut endoderm monolayer aggregation was reported [57]. This approach could remove a significant hurdle in the adoption of hPSC-derived GI organoid technology. This approach could also be used to generate gastric antrum and colon organoids. This research achievement made it possible to ship the starting material to other laboratories around the world, thereby significantly accelerating the use of human gastrointestinal organoids throughout medical research. In October 2022, Chen released important innovations in the high-throughput culture of liver organoids. The team developed a method to generate novel liver organoids by micropatterning [58]. This technique enabled the generation of homogeneous liver organoids, providing a powerful tool for liver drug screening and hepatotoxicity assessment.

Tumour organoids are established by 3D tissue culture of tumour stem cells in tumour tissues, which can simulate the characteristics of tumours in vivo and the heterogeneity of tumour cells. Cancer stem cells are also considered to be the producers of the entire tumour microenvironment [59, 60]. Currently, successfully cultured tumour organoids include those of colon cancer, gastric cancer, pancreatic cancer, prostate cancer, breast cancer, endometrial cancer, ovarian cancer, and kidney cancer. Circulating tumour cells (CTCs) are cells derived from primary tumours or tumour metastases. After shedding, CTCs enter the peripheral blood of patients through the blood system or lymphatic system, which is related to tumour invasion and metastasis [61, 62]. Therefore, organoids derived from CTCs are of great value for the study of tumour invasion and metastasis mechanisms. CTC-derived organoids have the advantages of a higher success rate, shorter generation time and better suitability for gene editing. Drug susceptibility testing of CTC-derived organoids can predict treatment outcomes in matched patients. However, CTC-derived organoids have only been established in a few cancers [62].

Most tumour organoid cultures currently use Matrigel hydrogel as a culture substrate [63]. Matrigel is a colloidal protein mixture secreted by Engelbreth-Holm-Swarm (EHS) mouse sarcoma cells and is produced by Corning Life Sciences [64]. The adjustment of tumour organoid induction medium composition is the most critical part of tumour organoid culture. Because tumour patients have individual differences, the induction factors of tumour organoids are not fixed, which also makes the development of commercial tumour organoid culture medium extremely difficult. The induction medium is the basal medium Dulbecco’s Modified Eagle Medium/Nutrient Mixture F-12 (DMEM/F-12) supplemented with various factors, and these factor components are key to the formation of tumour organoids. The various factors that need to be added to the medium must be explored and verified [65, 66]. These factors generally include the following four categories: (1) Wnt signalling pathway activators, which mainly promote angiogenesis and include Wnt signalling ligands and the GR5 ligand Rspo-1; (2) ligands of tyrosine receptor kinases, which mainly stimulate epithelial cell proliferation and include epidermal growth factor (EGF), recombinant fibroblast growth factor 10 (FGF10), and nerve growth factor (NGF); (3) TGF-β signalling pathway inhibitors, which mainly inhibit epithelial cell differentiation and include noggin, A83-01; and (4) ROCK inhibitors, which mainly maintains stem cell pluripotency and includes Y-27,632. The culture conditions of tumour organoids from different sources are different (Table 1) [67].

**Table 1:** Culture conditions for different tumor organoids

		Colorectal Cancer		Gastric Cancer	Breast Cancer	Pancreatic Cancer		Cholangiocarcinoma	Lung Cancer	Hepatocellular Cancer	Bladder Cancer	Esophagus Cancer	Thyroid Cancer	Prostate Cancer	Cervical Cancer	Ovarian Cancer	Head and Neck Cancer
Medium	Advance DMEM/F12	√	√	√	√	√	√	√	√	√	√	√	√	√	√	√	√
	HEPES 10 mM	√	√	√	√	√	√	√	√	√	√	√	√	√	√	√	√
	Glutamax 2 mM	√	√	√	√	√	√	√	√	√	√	√	√	√	√	√	√
	Antibiotics/antimycotics (1%)	√	√	√	√	√	√	√	√	√	√	√	√	√	√	√	√
Cytokines	rhR-Spondin-1 (ng/ml)	500	250	1000	250				500			500	250	125	250		100
	rhNoggin (ng/ml)				100	1000	100		100			100	25	100	100		40
	rmNoggin (ng/ml)	100	100	100													
	rhFGF basic (ng/ml)										12.5		20	1		50	5
	rhFGF-7 (ng/ml)				5				25		25				25		
	rhFGF-10 (ng/ml)			50	20	100			100	100	100	100		10	100		10
	rhIGF (ng/ml)															100	
	rhEGF (ng/ml)	50			5	50				50	10	50	20	50	50		50
	rmEGF (ng/ml)		50	50			50	50									
	rhWnt-3a (ng/ml)	50	50	25		50	25					50	50			20	
	rhVEGF121 (ng/ml)												10				
	rhNeuregulin 1 (nM)				5												
	rhHGF (ng/ml)									25							
Additive	N-2	1				1		1		1		1					
	N-21	1	1	1	1	1	1	1	1	1	1		0.5	1	1	1	1
Small Molecular Compounds	Wnt-C59						100										
	Y-27,632 (µM)				5			10	5	10	10		10	10	10	10	
	A83-01 (nM)	500	500	500	500	500	500	5000	500	5	5000	500	5000	500	500	500	
	Dihydrotestosterone (DHT) (nM)													0.1/1			
	Primocin													1:100v/v			
	Gastrin I (Human) (nM)	10	10	10		10		10		10						10	
	Forskolin (µM)							10		10					10		1
	Dexamethasone (nM)	10	10							3							
	SB 202,190				500 nM	10 mM	10 µM							10 µM	1 µM		
	Prostaglandin E2 (µM)								500								1
	Beta-Estradiol (nM)														100		
	Wnt Surrogate (nM)														0.5		
	CHIR 99,021 (µM)														0.3		3
	Nicotinamide (mM)	10			5	10		10	5	10	10	10	0.1	10	2.5		
	N-Acetylcysteine (mM)	1.25	1	1	1.25	1	1	1	1.25	1.25	1.25			1.25	1.25	1	1.25
References		[79]	[82]	[136]	[51]	[50]	[170]	[102]	[171]	[15]	[172]	[149]	[173]	[10]	[174]	[175]	[176]

After the organoid is established, it needs to be identified, and there are many methods to choose from (Fig. 2C) [15, 68]. According to the purpose of the research, the cost, and the feasibility of the identification method, the appropriate identification method can be selected. In the early stage of organoid culture, haematoxylin-eosin (HE) staining and immunohistochemistry are recommended to preliminarily determine the similarity between the cultured organoid and the source tissue [69]. If the morphological verification is qualified, expansion of the culture of organoids should be considered. Whole exome sequencing (WES) is then performed to identify the overlap rate of the characteristic genes between the organoid and the source tissue [70, 71]. If more accurate and comprehensive verification is needed, transcriptome sequencing, whole genome sequencing (WGS), and DNA methylation sequencing can be considered to analyse whether special targets, signalling pathways, or metabolic pathways are retained and to select qualified organoid models. To eliminate the misleading effect of cross-contamination on drug screening, short tandem repeats (STR) can be used to identify and verify that organoids are not contaminated by other cell lines or organoids during passaging [72]. Optimal growth organoids can be resuspended in cryopreservation medium and frozen at -80 °C or in liquid nitrogen (Fig. 2D) [73].

## Technological revolution in organoid models

To meet the different needs in cancer research, a number of important technologies and methods have been derived from the application of organoids. For example, researchers have established organoid biobanks for high-throughput and rapid screening of sensitive drugs. Organoid biobanks provide a powerful platform for discovering and screening anticancer drugs. To study the influence of immune cells on cancer cells, researchers have used cell coculture and chip technology to simulate the tumour immune microenvironment in vivo. Technological innovation has promoted the wider application of organoids in the field of cancer research.

### Organoid biobanks

Although the research results in tumours in recent years are extensive, these studies consume considerable manpower, material and financial resources to conduct research on each individual sample tissue. How to conduct high-throughput sample research and verification is an urgent problem in current cancer research.

With the widespread application of organoid technology, its clinical value has become increasingly prominent. Since organoids can be directly derived from diseased tissue (Patient Derived Organoid, PDO), it is feasible to further establish an organoid biobank [49, 74]. An established organoid biobank can provide drug susceptibility data for drug development research and help research in individualized medicine and regenerative medicine. It is also of great value to carry out related research on the dynamic occurrence, development and treatment of living tumours [75]. The main body of the tumour organoid biobank is organoids derived from tumour patients. Many organoids can be used at any time to carry out targeted scientific experimental research.

In 2015, Hans Clevers et al. established the first colorectal cancer organoid biobank. They successfully cultivated 22 colorectal cancer organoids from 27 surgically resected colorectal cancer samples, with an overall success rate of 90%. These organoids can be frozen after passage, and the survival rate after resuscitation can reach more than 80% [49]. Since then, several colorectal cancer biobanks have been established [76–83]. In 2017, the Hans Clevers team completed a large-scale breast cancer organoid project and established the world’s first breast cancer organoid biobank [51]. In 2018, Prof. Yan reported the establishment of a gastric cancer organoid biobank with associated genomic data, which provided a useful resource for studying gastric cancer cell biology and precision cancer therapy [74]. In 2022, Demyan L published an article on pancreatic cancer organoids in the journal *Annals of Surgery*. The researchers collected tumour samples from 117 pancreatic cancer patients, of which 16% were black, 9% were Asian, and 7% were Hispanic or Latino. This was the largest and most ethnically diverse pancreatic ductal adenocarcinoma (PDAC) organoid biobank reported thus far [26]. In August 2022, researchers established a library of 19 well-characterized and well-annotated paediatric rhabdomyosarcoma (RMS) organoids, which included all major subtypes of RMS. This was the first tumour organoid bank of pure mesenchymal malignancy (sarcoma) origin [84].

The establishment of tumour organoid biobanks is conducive to promoting the research progress of tumour molecular backgrounds and tumour biological behaviours (such as clinicopathological characteristics, drug response, etc.) and promoting the development of precision tumour treatment.

### Organoid immune cell coculture models

Suppression and reprogramming of the immune system play a key role in tumour initiation and progression [85–87]. The purpose of immunotherapy is to reactivate antitumour immune cells and overcome the immune escape mechanism of tumours. Immunotherapy works in several ways to help the immune system recognize tumours, initiate an immune response, or boost an existing immune response to fight tumour cells [12, 88]. As the tumour mutational burden varies among different tumours, tumour cells exhibit different immunogenicities, which leads to significant differences in immune responses. In recent years, the use of a patient’s own immune system to eliminate cancer cells in cancer treatment has achieved great success. However, a pressing problem in the field is the lack of suitable models to test and screen for this treatment. The emergence of organoids has ignited new hope for tumour immunotherapy.

A major limitation of organoids for testing the efficacy of immunotherapies is the lack of immune cell components. Immune cells in organoids are very different from those in the tumour immune microenvironment in vivo. At present, researchers are developing more complex organoid combination models, such as organoid immune cell coculture models [59, 60]. Organoid immune cell coculture models are mainly divided into two types. (1) One method is to retain and expand endogenous immune cells in tumour organoids or to add exogenous immune cells (autologous or allogeneic) [59, 89]. (2) Another method is the organoid chip, which can coculture tumour organoids and immune cells in a continuously perfused chamber by combining microfluidic technology and can reproduce the structural characteristics of the tumour microenvironment [90, 91].

Considering that tumour neoantigens may not bring the desired immune response, some scientists are trying to activate immune cells in vitro and then apply them to patients. In vitro activation of immune cells can be achieved using organoids. In September 2018, the journal *Cell* published a study by Professor Dijkstra KK [92]. They established patient-derived tumour organoids and simultaneously extracted peripheral blood lymphocytes from patients with colon or lung cancer. By coculturing CD8^+^ T lymphocytes with tumour organoids to activate immune cells, T cells with antitumour activity were screened. The treated killer T cells showed a good tumour killing effect in in vitro cell experiments. The key tool for this was tumour organoids. Without tumour organoids, it was impossible to produce specific killer T lymphocytes. Although this study demonstrated that organoid-induced T cells kill tumours in vitro, their role in vivo remains unclear. Therefore, large-scale clinical studies are needed to verify these findings [92]. Alternatively, circulating lymphocytes or tumour-infiltrating lymphocytes (TILs) are collected, screened, modified, expanded, and activated in vitro and then reinjected back into the patient for cancer treatment. Michie J and Schnalzger TE studied chimeric antigen receptor T-cell (CAR-T) and natural killer cell (NK cell)-mediated cytotoxicity in tumour organoid models, confirming that organoids are an effective platform for evaluating CAR cell efficacy and tumour specificity [93, 94].

Michie J established human epidermal growth factor receptor-2 (HER2)-expressing colorectal cancer organoids and added anti-HER2 CAR-T cells for treatment [93]. CAR-T cells alone caused only weak killing, while the combination of CAR-T cells and the apoptosis antagonist birinapant significantly increased the death of tumour organoids. This combination therapy could also initiate potent tumour-killing effects. Colorectal cancer organoids were also used to track CAR-mediated cytotoxicity in NK cells in a study by the Schnalzger TE research group. Using a colorectal cancer organoid model, the researchers demonstrated efficient targeting of CAR-NK cells against the ubiquitous epithelial antigen epithelial cell adhesion molecule (EpCAM) [94]. Specific CAR-NK cells effectively killed tumour organoids expressing EGFRvIII but not normal tissue organoids. PDTOs could be used to monitor and enhance the recruitment and infiltration of immune cells into tumour tissues and serve as a platform to evaluate the efficiency of tumour-specific T-cell killing.

Recently, Carine Bouffi successfully developed a new generation of complex intestinal organoids that include key elements of a functional immune system. This is the first in vivo organoid containing a functional immune system that scientists have developed thus far [95]. These results indicated that organoids have broad application prospects in tumour immunotherapy.

### Organoids-on-chip

Organoids-on-chip technology combines organoids and chips and integrates the advantages of organoids and organ chips [96]. An organ physiological microsystem is built on a chip, which can be used to predict the body’s response to drugs or different external stimuli. Mature organoid chips can almost simulate the microenvironment of the human body. Studies have shown that organoids-on-chips have great advantages over ordinary organoid models. In August 2022, the Food and Drug Administration (FDA) approved the world’s first new drug based entirely on organoid-on-a-chip research and obtained preclinical data to enter clinical trials (NCT04658472). This milestone means that in vitro drug testing through the organoid-on-chip platform will replace the traditional in vitro model and is increasingly used in the development of new drugs [97].

The organoid-on-a-chip is constructed directly from human tissue. Thus, the drug effect can be more fully evaluated before clinical trials, and inappropriate drugs can be excluded, thereby improving the success rate of drug development. Organoids-on-chip address the shortcomings of traditional organoid applications, such as the lack of immune cells and vascular structures, and these advantages are crucial for research in the field of tumours [96]. Organoids-on-chip integrate microfluidic technology, which can control the time of exogenous substance addition [98]. Shirure et al. established a tumour organoid-on-a-chip that can mimic angiogenesis of endothelial cells and support the construction of breast cancer patient-derived organoid blood vessels [99]. Although this process was distinct from vascularization during embryogenesis, it could also help characterize the tumour microenvironment in vivo and maintain organoid growth. Microengineering techniques incorporated in organoid chips could assist organoids in mimicking the various types of mechanical forces experienced during real development, which are integral to regulating organ development and maturation. The platform mimicked the intraluminal flow and rhythmic contractions of the stomach in vivo by using a peristaltic pump attached to a pipette [100].

Organoids can simulate the interaction between different tissues and different organs by using microfluidic arrays to coculture organoids from different tissue sources. Jin Y et al. established a multichamber microchip device for culturing hepatocytes and human endothelial cells. By simulating blood circulation in vivo using a laboratory rocker, vascularized liver organoids were generated after 21 days [101]. The researchers also cocultured liver, intestinal, and gastric organoids and allowed the different culture chambers to communicate through media flow. The results showed that paracrine factors produced by intestinal organoids reduced the expression of bile acid synthase in liver organoids, revealing the interactions between physiological organs [101].

Organoids-on-chip play an important role in the fields of new drug development, personalized treatment and regenerative medicine by combining organoid technology and organ chip technology. However, due to technical limitations, organoids-on-chips still have many limitations. With the development of technology and the integration of multiple disciplines, it is believed that organoids-on-chip can better contribute to human health in the future.

## Application of organoids in cancer research

**Fig. 3:**
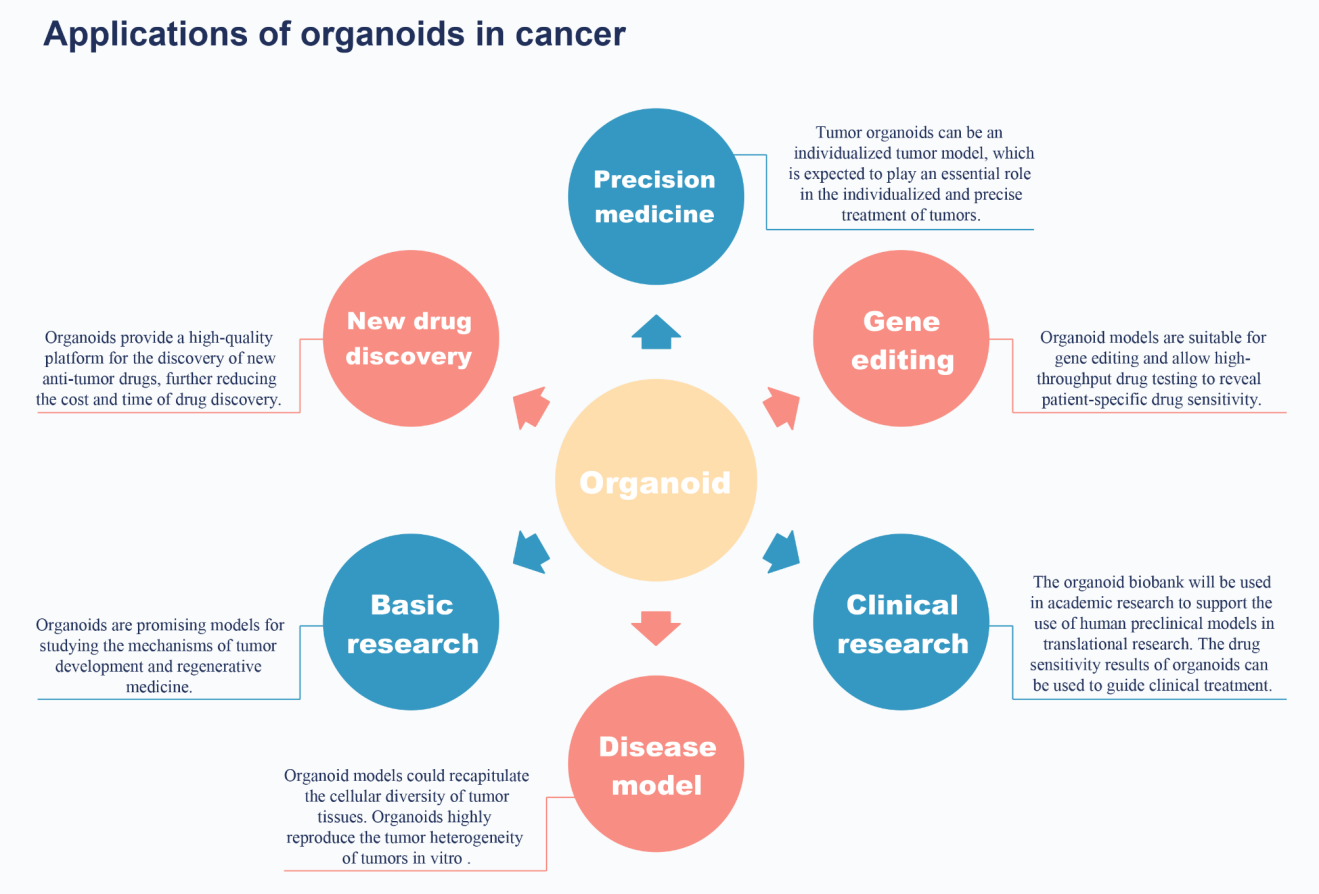
The application of organoids in cancer is mainly reflected in six aspects: precision medicine, gene editing, clinical research, disease modelling, basic research, and new drug discovery

Organoids are of great value in cancer research. Organoids play an important role in disease models, precision medicine, new drug discovery, gene editing, and basic and clinical research (Fig. 3).

Organoids are promising models in vitro, and they provide a platform for basic and clinical research on cancer. In February 2018, *Science* published a study using tumour organoid models to predict tumour responsiveness to anticancer drugs. It was found that the tumour organoid model predicted patient response to drugs with an overall sensitivity of 100%, a specificity of 93%, a positive predictive value of 88%, and a negative predictive value of 100% [80]. In short, we can know ahead of time which treatments are not working for patients, freeing up valuable time to try treatments that might work.

Tumour organoids are most commonly used in drug sensitivity screening to guide individualized precision medicine approaches for cancer treatment [102, 103]. Drug sensitivity testing of tumour organoids is similar to that of cell lines cultured in vitro. Both chemotherapy drugs and most targeted drugs can be tested, and both single drugs and drug combinations can be examined. However, not all chemotherapeutic drugs are suitable for drug sensitivity testing with tumour organoids. These drugs include (1) chemotherapeutic drugs that need to be metabolized in vivo to produce antitumour active ingredients, such as capecitabine and irinotecan. They also include (2) certain targeted drugs, such as antiangiogenic targeted drugs (axitinib), that act through vascular endothelial growth factor receptor (VEGFR)-1/2/3 in vascular endothelial cells, as tumour organoids cultured in vitro do not contain vascular structures. Another drug class that is not suitable is (3) immunotherapy drugs, such as immune checkpoint inhibitors, as their function requires the participation of the tumour immune microenvironment in vivo.

Organoids are of great value for the development of new drug discovery [104, 105]. Using organoids in the early stages of drug development can help identify those drugs that work for most patients and even for tumours with specific mutations. Additionally, using organoids from healthy tissue can identify side effects of drugs on the organ [106, 107]. One of the main reasons for drug failure in clinical trials is liver toxicity, and hepatocyte organoids can be used to test the liver toxicity of potential new drugs [108]. However, the use of organoid drug sensitivity screening to guide clinical treatment is in its infancy, and a large number of clinical studies are still needed to confirm its reliability and safety.

Organoids can be genetically engineered using CRISPR‒Cas9 gene editing technology to study the effects of specific oncogenic mutations controlled by alleles. In vitro models of colorectal cancer were induced by introducing mutations in knowledge representation for autonomous systems (KRAS), adenomatous polyposis coli (APC), recombinant mothers against decapentaplegic homologue 4 (SMAD4), and tumour protein p53 (TP53) in wild-type intestinal organoids [109, 110]. Stephen Meltzer cultivated a gastro-oesophageal junction (GEJ) organoid from healthy human tissue and used CRISPR‒Cas9 gene editing technology to knock out two key tumour suppressor genes, TP53 and Cyclin Dependent Kinase Inhibitor 2 A (CDKN2A), thus revealing the mechanism of early GEJ tumour development [111].

Organoids have been used in different tumour types (Table 2). Herein, we describe the current status of organoids in the field of tumour research according to the time sequence in which tumour organoids were successfully established.

**Table 2:** The time of the first report of different tumor organoids

Sources of Tumor Organoids	First Report Time	Publication	References
Colon	2011	*Gastroenterology*	[48]
Prostate	2014	*Cell*	[10]
Pancreas	2015	*Cell*	[50]
Brain	2016	*Cancer Research*	[122]
Liver	2017	*Nature Medicine*	[15]
Endometrium	2017	*Nature Cell Biology*	[133]
Stomach	2018	*Science*	[80]
Breast	2018	*Cell*	[51]
Bladder	2018	*Cell*	[20]
Esophagus	2018	*Nature Communications*	[149]
Lung	2019	*Nature Communications*	[152]
Rectal	2019	*Nature Medicine*	[79]
Kidney	2019	*Cell Death & Disease*	[158]
Biliary tract	2019	*Cell Reports*	[102]
Ovary	2019	*Nature Medicine*	[11]

### Gut

Colon cancer organoids were the first successfully established tumour organoids. In 2011, these organoids were first reported by Professor Hans Clevers, the originator of organoids [48]. Since then, colon cancer organoids have become an important model for studying the evolution of colon cancer. In 2017, Toshiro Sato confirmed that LGR5 + tumour cells have the characteristics of tumour stem cells, revealing their self-renewal and differentiation capabilities [112]. Knockout of LGR5 + cells leads to transient tumour regression, but KRT20 + tumour cells quickly differentiate into LGR5 + tumour cells. These cells exert the characteristics of tumour stem cells and continue to promote tumour growth. Mark Schmitt’s research using colorectal cancer organoids has revealed the direct impact of dying cancer cells on adjacent tumour epithelial cells and elucidated the molecular mechanisms that lead to treatment resistance through paracrine effects [113]. The research results also create new opportunities for the treatment of tumour drug resistance. Using colorectal cancer organoids, Dmitrieva-Posocco O demonstrated that β-hydroxybutyrate (BHB) increased HOPX expression and inhibited the growth of patient-derived colorectal cancer organoids [114]. This study provides a new target for the treatment of colorectal cancer and has important clinical value.

The rectum and colon are highly similar in structure, but there are still some differences in molecular biological characteristics. Therefore, it is more suitable to establish rectal cancer organoids for rectal cancer research. In 2019, Smith, JJ established 65 tumour organoids from patients with primary, metastatic or recurrent rectal cancer. The established rectal cancer organoids retained the molecular characteristics of the source tumours, and the results of in vitro drug sensitivity experiments correlated with the clinical responses of tumour patients [79]. Guoqiang Hua found similar histopathological features between primary rectal cancer and rectal cancer organoids, and organoids showed a sensitivity of 78% and specificity of 92% in predicting the clinical response of patients. The study elucidated the strong correlation between the chemoradiotherapy sensitivity of patient-derived tumour organoids and the clinical responses observed in large cohort studies [77]. In short, colorectal cancer organoids are one of the earliest and most mature organoids and have been widely used in clinical and basic research on colorectal cancer. Thus, important research results are emerging continuously, and these organoid systems present good application prospects.

### Prostate

Prostate cancer is a malignant tumour with high incidence in older men. Gao D’s research was the first to report that organoids derived from human prostate tumours can be grown in the laboratory. The researchers successfully generated six prostate cancer organoids and one organoid derived from circulating tumour cells. The tissue structure of these prostate cancer organoids was highly similar to that of the metastatic samples from which they originated [10]. The research results were published in *Cell* in 2014. In 2016, Hans Clevers published a culture protocol for generating 3D prostate organoids, which can be obtained in approximately 2 weeks. The prostate cancer organoids established according to this protocol highly imitate the characteristics of tumours in vivo and can be widely used in molecular mechanism research and drug screening for prostate cancer [115].

Zhang Z found that cancer-associated fibroblasts (CAFs) promote anti-androgen resistance in prostate organoids. Following blockade of the NeuReGulin-1/human epidermal growth factor receptor-3 (NRG1/HER3) axis using antibodies, tumours were resensitized to antiandrogen therapy [116]. Sawyers CL used mouse prostate organoids to study the biological characteristics of wild-type forkhead box protein A1 (FOXA1) and mutant FOXA1. Studies have confirmed that mutations in FOXA1 alter its function, disrupt the normal luminal epithelial differentiation program, and further promote prostate cancer progression [117]. In the future, it is hoped that prostate cancer organoids will bring new breakthroughs in the diagnosis and treatment of prostate cancer.

### Pancreas

Pancreatic cancer is one of the tumours with the worst treatment effect and prognosis among digestive system tumours. Pancreatic cancer organoids were first established by the team of Professor Boj SF [10]. The research results were published in *Cell* in 2015. The researchers extracted normal and tumour tissues from mouse and human pancreases, respectively, and successfully established normal and tumour pancreatic organoids by modifying the previous method of culturing gastrointestinal tumour organoids. The success rate of creating pancreatic cancer organoids was approximately 80%. This study opened a new chapter in organoid research in pancreatic cancer.

In April 2021, *Cell Stem Cell* published two articles on pancreatic cancer organoids [118, 119]. Both studies used pluripotent stem cells (PSCs) that differentiated into pancreatic progenitor cells (PP) and then formed pancreatic organoids in Matrigel. The same oncogene mutations were then manipulated to induce the formation of pancreatic cancer organoids. The Harvard study created both pancreatic ductal and acinar organoids. These two studies provide detailed strategies for the establishment of pancreatic cancer organoids. In October 2021, Barbara T. Grünwald used pancreatic ductal carcinoma organoids to reveal the role of different mesenchymal subtypes (deserted, reactive, and intermediate) in tumour development and response to drug therapy [120]. They established pancreatic ductal carcinoma organoids and cocultured them with CAFs from different stromal subtypes. The results showed that reactive tumour stroma could promote tumour cell proliferation and shorten disease-free survival. This study clarified the mechanism of pancreatic cancer chemotherapy resistance and provided a new direction for the treatment of pancreatic cancer [120]. In the same year, Jorgensen, C. published a study revealing the role of the laminin-integrin α3/α6 signalling pathway in the establishment and survival of pancreatic organoids, establishing a more ideal pancreatic cancer organoid. It can better reproduce the characteristics of the tumour microenvironment and is more suitable for basic and translational research on pancreatic cancer [121].

At present, the establishment scheme of pancreatic cancer organoids has been continuously improved, and an increasing number of organoids are being used in clinical and basic research on pancreatic cancer. Pancreatic cancer organoids have brought new hope to overcome difficult problems, such as pancreatic cancer chemotherapy resistance.

### Brain

Brain tumours are among the most aggressive and least survivable types of human tumours. In 2016, the team of Rich, JN reported brain tumour organoids for the first time [122]. The researchers created tumour organoids in tissue culture from glioblastoma. The biological characteristics of patient tumour-derived organoids are similar to those of parental tumours. Since then, brain tumour organoids have opened a new chapter in brain tumour research.

Brain tumour organoids have been widely used in clinical and basic research [123–127]. In 2018, Shan Bian established a brain tumour organoid model. The researchers claim that this model can reproduce the complex structure of the human brain and the occurrence of brain tumours in vitro and can be used for clinical drug screening. The study successfully screened 18 single-gene mutations and 15 mutation combinations that are considered to be critical for brain tumorigenesis using brain tumour organoid models [128]. Davy, A focused on the role of dihydrofolate reductase (DHFR) in brain tumours. The authors used brain tumour organoids and found that methotrexate can inhibit DHFR, reduce the self-renewal ability of brain tumour organoids, and inhibit the tumorigenic ability of brain tumour initiating cells (BTIC) [129]. In conclusion, brain tumour organoids provide a platform for rapid screening of oncogenes and screening of effective drugs in vitro, bringing new hope to the research of brain tumours.

### Liver

Liver cancer is one of the most common malignant tumours. Humans have been devoted to research on liver cancer for more than half a century. There is an urgent need to identify the underlying mechanism of liver cancer progression and explore new therapeutic drugs. A study published in the journal *Nature Medicine* in 2017 brought new hope to liver cancer research [15]. Researchers successfully grew the first human primary liver cancer organoid model in the laboratory and used it to test 29 different cancer drugs. This research achievement was regarded as a major milestone in the development of liver cancer research [15]. In 2018, Nuciforo, S used patient biopsy tissue to construct liver cancer organoids for drug sensitivity testing [130]. The researchers used tumour organoids to conduct drug sensitivity tests on sorafenib and found that organoids from different patients had different sensitivities to sorafenib. The results of the study confirmed that the organoid model derived from the biopsy tissue was highly consistent with the parental tumour tissue, which can provide a tool for the development of individualized precision therapy [130].

In 2019, researchers used CRISPR gene editing technology to alter the genes of normal human liver organoids to explore the role of a certain gene mutation in tumour formation. Using this approach, the researchers found that BAP1 mutations altered organoid behavioural traits, such as faster growth and easier fusion. These features resemble more aggressive malignancies [131]. In early 2023, Bin Li reported using tumour organoids derived from liver cancer patients to screen 419 FDA-approved drugs and found that loratadine could inhibit the growth of liver cancer organoids [132]. This study used tumour organoid models to confirm that desloratadine may be a new type of anticancer drug, and NMT1 is expected to become a potential biomarker and therapeutic target for liver cancer.

### Endometrium

Endometrial cancer is a malignant tumour that occurs in the endometrium. It is one of the three major tumours of the female reproductive system and accounts for 20-30% of these tumours. In 2017, Turco, MY established three-dimensional cultures of normal and decidualized human endometrium by adapting the conditions used to establish human adult stem cell-derived organoid cultures. The researchers also grew organoids from malignant endometrium for the first time [133]. These organoids are capable of long-term expansion, providing an in vitro model for the study of endometrial cancer. In the same year, Girda, E reported the establishment process of endometrial cancer patient-derived organoids, which were used to screen various drugs, including endocrine therapy drugs. Studies have confirmed that organoids are very similar to the source tumours in histomorphology and immunohistochemical expression [134]. Therefore, in vitro drug susceptibility testing using endometrial cancer patient-derived organoids is feasible. In 2019, Boretto, M reported the establishment of organoids extracted from low- to high-grade endometrial cancer patients for in vitro drug screening [17]. In the future, these organoids may replace patient-derived xenograft (PDX) mouse models, significantly reducing the overall cost associated with in vivo models [17, 135].

### Stomach

Gastric cancer is a common malignant tumour of the digestive tract. Gastric cancer organoids were first established by Vlachogiannis G., and the research was published in *Science* in 2018 [80]. The genotypes of the gastric cancer patient-derived organoids established by the researchers were highly similar to the original patient tumours, and the molecular analysis of the tumour organoids highly matched the drug screening results [80]. These research results provide a new in vitro model for clinical and basic research on gastric cancer. In August, a research team established a biobank of gastric organoids. The library consists of genetically engineered gastric organoids carrying various mutations and 37 patient-derived organoids. This study details the characterization of human gastric cancer organoids derived from patients with different tumour subtypes. The results revealed the key pathways of gastric carcinogenesis, emphasizing the important value of genotype-phenotype screening strategies for an in-depth understanding of gastric cancer [136]. In December, the team of Professor Yan, H.H.N. reported the establishment of a primary gastric cancer organoid biobank, which contained normal, abnormal hyperplasia, cancer and lymph node metastasis samples from 34 patients [74]. Tumour organoids are highly similar to the source tumour in terms of morphological characteristics, genome profile and transcriptome profile and can be used for large-scale drug screening. Thus, they can serve as high-quality resources for molecular mechanism research and individualized precision treatment of gastric cancer. Since 2018, gastric cancer organoids have been gradually applied in basic and clinical research on gastric cancer, and research results have continued to emerge.

In 2019, human gastric cancer organoids were established by the team of Seidlitz, T. team. These organoids simulated the typical characteristics and pathway variations of human gastric cancer and confirmed the application value of gastric cancer organoids in studying molecular mutation patterns and drug sensitivity [137]. The study found that trastuzumab can be used to target organoids carrying HER2 mutations, and palbociclib can be used to target organoids carrying CDKN2A deletion mutations. Gastric cancer organoids can be used not only to identify effective drugs but also to determine which drugs are resistant. Thus, they can be used to reveal drugs that only cause side effects but have little therapeutic effect and should therefore be avoided in clinical treatment [137]. In addition to being used in drug screening, gastric cancer organoids can also be combined with technologies such as CRISPR/Cas9 editing to explore the characteristic phenotypes and functions of different mutation types of gastric cancer. In 2021, Yuan-Hung Lo used primary human gastric organoids combined with gene editing technology to establish the first genetic model of ARID1A mutations, and its multiomics analysis revealed many characteristic phenotypes of ARID1A mutant gastric cancer [138]. The results of the study found that the loss of ARID1A caused mucinous metaplasia, inhibited Wnt/β-Catenin pathway activity, and promoted tumorigenesis in primary human gastric organoids [138]. Human tumour organoids edited by CRISPR/Cas9 technology provide a valuable platform for studying human gastric cancer development. Gastric cancer organoids have good application prospects in the future and are effective tools for research on the molecular mechanisms of gastric cancer occurrence and evolution, biomarker identification, drug screening, and preclinical evaluation of individualized drug strategies [139].

### Breast

In January 2018, Hans Clevers first reported a method for long-term culture and maintenance of breast epithelial organoids, and the success rate of breast cancer organoid culture was over 80% [51]. The team successfully established more than 100 breast cancer primary tumour and metastatic tumour organoids. They also conducted in-depth histopathological and gene sequencing analysis on the organoids and conducted in-depth research on the drug sensitivity of breast cancer organoids. Breast cancer organoids well maintain the characteristics of the original tumour in histopathological morphology. In February of the same year, an article published in *Nature Methods* introduced how to use the breast cancer susceptibility gene (BRCA) mutation mouse model of breast cancer to establish organoids and used this model for therapeutic drug research [140]. The research team that published the article is from the Netherlands, and Hans Clevers is also on the list of authors. The P53 and BRCA1/2 genes are well-known tumour suppressor genes, and their mutations can cause tumour syndromes, one of which is breast cancer. This study described the establishment method of P53/BRCA gene mouse breast cancer organoids and confirmed the application value of breast cancer organoids in breast cancer research.

Breast cancer organoids provide a good model for molecular mechanism research and clinical drug screening of breast cancer. Cong, M used breast cancer organoids to explore the molecular mechanism of metastasis suppressor 1 (MTSS1) [141]. Xiaolong Wang used patient-derived organoids to demonstrate that targeting circRNA-CREIT could serve as a promising therapeutic strategy for chemotherapy-resistant triple-negative breast cancer [14]. Chen Ping established a breast cancer organoid biobank that contains tumour tissues of different disease stages and molecular subtypes [142]. Patient-derived breast cancer organoids can serve as a diagnostic platform to support and guide drug therapy in advanced breast cancer. There are several different molecular subtypes of breast cancer, and there are significant differences in the genomic variation among the various subtypes, which is the basis for the choice of treatment options in advanced breast cancer. Breast cancer organoids provide a good platform for the in-depth exploration of the differential characteristics of different subtypes, which is of great significance in breast cancer precision medicine research.

### Bladder

Bladder cancer is a malignant tumour originating from the urothelium of the bladder, and it ranks first in the incidence of urinary system tumours. In 2018, the team of Professor Michael M. Shen successfully cultivated bladder cancer organoids for the first time, and the research results were published in the journal *Cell* [20]. Researchers isolated tumour cells from bladder cancer of 22 patients and cultured them into organoids with a diameter of approximately 1 mm in vitro [20]. These tumour organoids shared the same molecular and genetic characteristics as the patient’s actual tumour. The researchers also plan to conduct clinical trials to test the accuracy of bladder cancer organoids in predicting treatment response.

Bladder cancer organoids are increasingly used for tumour biology research and drug screening [143–147]. Tan, P reported organoid-based small molecule drug screening for bladder cancer for the first time [144]. The study found that the Sirtuin1 (SIRT1) activator SRT1720 significantly inhibited the growth of mouse and human bladder cancer organoids. Another research team reported the use of patient-derived bladder cancer organoids to evaluate CAR-T-cell-mediated cytotoxicity against bladder cancer cells [148]. The research team successfully cultivated two main subtypes of bladder cancer organoids using the self-developed bladder cancer organoid culture system. Immunofluorescence staining experiments confirmed that the staining results of molecular markers of bladder cancer organoids and their source tumour tissues were consistent, which indicated that the biomarkers in cancer tissues were well reproduced on bladder cancer organoids. To evaluate the application of bladder cancer organoids in testing the killing effect of CAR-T cells, researchers cocultured bladder cancer organoids with mucin 1 (MUC1)-CAR-T cells. The results showed that the expression levels of interleukin-2 (IL-2), tumour necrosis factor α (TNF-α), and interferon γ (IFN-γ) were all significantly increased. These results fully demonstrated the potential application value of bladder cancer organoids in evaluating the killing effect of CAR-T cells [148].

### Oesophagus

In 2018, a research team reported the successful establishment of 10 oesophageal adenocarcinoma organoid models [149]. In this study, the medium of gastric cancer organoids was used to culture oesophageal cancer organoids. However, only 10 of the 31 samples were successfully established as organoids, and 9 of them could be passaged for more than half a year. The author analysed the possible reason that most of the experiments failed and found that it was due to an insufficient amount of initial tumour cells. Genomic analysis revealed that oesophageal cancer organoids are as polyclonal as the original tumour. A more interesting finding is that organoids are not static during subculture. Additionally, some subclones gradually become dominant clones because they are better adapted to the environment, resulting in changes in the genomics of organoids [149]. This discovery is of great value to the study of tumour evolution and tumour drug resistance. This study confirmed the potential value of using organoids for individualized tumour drug sensitivity screening and demonstrated the application prospects of tumour organoids in oesophageal cancer research. The 3D oesophageal cancer organoid model has also been used to study the molecular mechanism of oesophageal cancer [150, 151]. Professor Tang, Q. ‘s team used oesophageal cancer organoids to find that the knockdown of the Rab11-FIP1 gene led to increased organoid size. Loss of Rab11-FIP1 increases tumour cell invasion, partly through mutated p53 but also in an independent manner [151]. The researchers cultured organoids from stem cells in the patient’s own normal tissue and then used CRISPR/Cas9 technology to knock out two key tumour suppressor genes (TP53 and CDKN2A) in the organoids. Knocking out these genes made the cells more cancerous, and they rapidly grew into malignancies. This model may be used to further our understanding of the early occurrence and progression of gastroesophageal junction carcinogenesis and reveal its potential therapeutic strategies [111].

### Lung

In 2019, Kim, M. first reported lung cancer organoids [152]. In this study, different types of primary lung cancer tissue and normal bronchial tissue were used as samples for organoid culture, and then the cultured organoids were compared with the primary tissue for histology, morphology, and genomics. Studies have confirmed that lung cancer organoids are highly consistent with primary tumour tissues. Lung cancer organoids were used for drug sensitivity tests of docetaxel, erlotinib, crizotinib, and olaparib, and finally, the PDX model was used to verify the drug screening results of lung cancer organoids [152]. Drug screening using lung cancer organoids is highly consistent with gene sequencing results, and the results are more accurate and intuitive. Afterwards, several laboratories successively reported the successful establishment of lung cancer organoids and conducted related research [153–156]. In January 2023, Yilong Wu published the largest research cohort of lung cancer organoids in the world. The main aim of the study was to predict the efficacy of locally advanced or metastatic lung cancer using patient-derived organoids [157]. The researchers established 212 lung cancer organoids from 107 patients, with a success rate of 81.5%. Based on the accuracy of the drug sensitivity test results of lung cancer organoids to predict clinical efficacy, the results confirm that lung cancer organoids have the ability to predict the effectiveness of treatment options. Therefore, lung cancer organoids are a promising tool for precision medicine.

### Kidney

Renal cell carcinoma (RCC) is a malignant tumour originating from the epithelium of the urinary tubules of the renal parenchyma. In 2019, Bonci D. first reported the establishment of kidney cancer organoids and established 10 kidney cancer organoids from 15 samples [158]. For the first time, this study confirmed the future prospects and application value of kidney cancer organoids.

In 2020, a study reported the establishment of the first childhood kidney cancer organoid biobank, providing an impetus for childhood kidney cancer research [159]. Single-cell RNA sequencing analysis revealed that organoids from different patients had distinct cell populations and distinct cell subpopulations within the same organoid. Thus, Wilms tumour organoids retained the cellular heterogeneity of the disease. The organoids were highly similar to their corresponding tumour tissues. They retained the epigenetic characteristics of the original tumour, and serial passages did not disturb the genetic stability of organoids. The researchers used childhood renal cancer organoids for drug sensitivity testing, and the results showed that childhood renal cancer organoids can be used for high-throughput drug testing to reveal patient-specific drug sensitivity [159]. In 2022, Xue, W. established 7 strains of kidney clear cell carcinoma organoids and conducted a drug sensitivity test on toripalimab (a PD-1 inhibitor), confirming that tumour organoids are a promising and reliable preclinical model for predicting the response to immunotherapy in RCC [160]. In the same year, Weiren Huang’s study also confirmed that patient-derived RCC organoids were valuable preclinical models for academic research and personalized medicine [161].

## Challenges and future perspectives

As a 3D cell culture model, organoids are highly similar to human organs in structure and function and have the characteristics of cell proliferation and differentiation, self-renewal, self-assembly, long-term culture, and genetic stability [162]. A number of studies have confirmed that organoids have a high degree of clinical relevance and can efficiently carry out drug sensitivity testing for patients, making individualized precise tumour treatment possible [6, 163]. Compared with established cell lines, cell line-derived xenograft (CDX) and PDX models, tumour organoids are irreplaceable as disease models (Table 3). However, we must also acknowledge that the development of organoids is still at an early stage, and there are still many limiting factors restricting the application of this technology.

**Table 3:** Comparison of advantages and disadvantages of common cancer models

	Cell model			Animal model			Organoid model
	Primary cells	Cell lines		Cell-derived xenograft	Patient-derived xenograft		
**Methods**	Cells isolated directly from animal or human tissue.	Cells that converge in function, metabolism, and morphology. Infinite proliferation and immortalization.		The tumour cells cultured in vitro were inoculated subcutaneously into immunodeficient mice.	Patient-derived tumour tissue was implanted into immunodeficient mice.		Derived from embryonic stem cells or induced pluripotent stem cells (iPSCs). Derived from tumour tissue of patients.
**Advantages**	Similar characteristics to animal or human cells.	Less interference factors, easy synchronization, easier control of experimental conditions and easy gene manipulation.		The effect on the host is similar. Tumour morphology, growth rate, drug sensitivity, and death time of animals were very similar.	Preserve the microenvironment of parental tumour growth. High tumour similarity. Preserve tumour heterogeneity.		Simulate the complexity of tumour microenvironments. High plasticity. The cultivation time is short. There are no ethical issues.
**Disadvantages**	Poor uniformity. The proliferative ability is low and cannot be passaged. The transfection efficiency is low.	Partial or complete loss of the characteristics of primary cells. Mutations may occur during long-term passage.		The growth rate is fast, the proliferation ratio is high, and the volume doubling time is short, which is significantly different from human tumours.	The in vivo microenvironment cannot be fully simulated. Model building takes a long time. The success rate of model building is low.		Lack of innate immune cells. No endocrine and neural regulation. The technology is not yet mature.

The reproducibility and consistency of organoid culture are the first problems to be addressed that largely affect the development of organoids. At present, there is no standardized process for organoid harvesting, culture, cryopreservation and recovery, and there is a lack of established industry standards. There are many human factors involved in the establishment of organoids, resulting in differences in the organoids established by different researchers. Even organoids created by the same researcher in different batches varied in shape and function [164]. To address this problem, it is necessary to continuously explore the optimal cultivation conditions and formulate recognized standards. It is also necessary to improve the automation level of the organoid establishment process and reduce the interference of human factors. In addition, organoid identification technology is important for ensuring good repeatability and consistency. However, the current observation of organoids is mainly focused on morphology, and technology to detect various indicators of living organoids in real time is still relatively lacking.

Currently, mouse-derived extracellular matrix (ECM) substitutes and foetal bovine serum are commonly used as culture media. Matrigel is difficult to apply to many human treatments because it contains exogenous components. Compared with the human physiological environment, organoids lack connective tissue, blood vessels and immune cells [162, 164]. To address this issue, the main methods used by researchers are techniques such as coculture and microfluidics. In 2020, the journal *Nature Protocol* published a protocol for the coculture of tumour organoids and immune cells, which can simulate some characteristics of the tumour microenvironment and the interactions between tumours and immune cells [165]. Although some studies have reported the combined application of organoids and microfluidic technology, microfluidic technology is still immature. Moreover, engineering control of the organoid culture process is still an urgent problem to be addressed. In addition, the diameter of existing organoids is approximately 100–500 μm. Although this size range has a certain degree of scale effect, it is still difficult to simulate real tissues and organs. If larger organoids are to be produced, the vascularization of organoids is also a problem that needs to be addressed.

Although patient-derived tumour organoids can reflect some tumour heterogeneity, patient-derived tumour organoids cannot fully reproduce the tumour microenvironment [166]. Some tumours, such as gastric cancer, have high tumour heterogeneity. Thus, gastric cancer organoids cannot fully reflect the heterogeneity of gastric cancer tissues in the body, which will reduce the efficiency of drug screening for gastric cancer organoids [164]. The current method to address this issue is to take tumour tissues at multiple points to build organoids, but this still cannot fully reflect the heterogeneity of tumours in vivo. As a new type of drug screening model, organoids are more cost effective than PDXs but are still more expensive than cell lines. Compared to the culture of cancer cell lines, the cultivation of organoids requires more time and resources. Organoids do not have an absolute advantage over other models in any aspect, so comprehensive judgement should be used to decide the best method.

Recently, a study reported by Professor Carine Bouffi was the first attempt to establish intestinal organoids in humanized mice. The team successfully obtained intestinal organoids with immune cells, opening the era of next-generation organoids [95]. From the 16th week, human intestinal organoids already have the typical signs of embryonic intestinal immune development, such as the accumulation and partitioning of T cells and B cells and the infiltration of granulocytes and plasma cells. This study overcomes the limitation of organoids lacking an immune microenvironment and opens a new platform in the field of immunotherapy of organoids. It is expected to bring new opportunities for tumour immunotherapy in the future.

Although there are many difficulties in the road ahead, we still have hope for the future of organoid research. Researchers are paying more attention to organoids than ever before. It is believed that in the near future, industry standards for various organoids will emerge. The combination of organoids and new technologies has continuously broken through application limitations and brought novel advancements to research. Examples include the combination of organoids with in vivo real-time imaging technology, 3D bioprinting technology, and the “Human Cell Atlas (HCA)” [167–169]. As a highly simulated disease model, organoids are expected to continue to make new progresses in precision medicine, regenerative medicine and other fields. We have reason to expect that organoids will play a greater role in the field of biological and clinical medical research in the future.

## Conclusion

The development of organoid technology has provided a new cancer model. Tumour organoids can simulate the structure and function of tumours in vivo to the greatest extent and are more suitable for in vitro tumour biology and treatment-related research. With the continuous deepening and progress of organoid research, the current bottlenecks that need to be overcome will be addressed; such solutions include further optimizing conditions to improve the timeliness of detection, improve the success rate of culture, simulate organoid vascularization, and simulate the tumour immune microenvironment. The response of organoids to drugs is highly correlated with patient clinical data, providing a reliable model for cancer research and treatment. In the future, organoids will complement other models and technologies and will open up new horizons for new drug development, drug screening, tumour mechanism research, and personalized precision therapy.

## Data Availability

Not applicable.

## Declarations

### Abbreviations

Three-dimensional (3D); Patient-derived tumor organoids (PDTOs); Extracellular Matrix (ECM); Engelbreth-Holm-Swarm (EHS); Mesenchymal stem cells (MSC); Induced Pluripotent Stem Cells (iPSCs); EGF, Noggin, R-spondin (ENR); Colorectal cancer (CRC); Embryonic stem cells (ESCs); Adult stem cells (ASCs); Human pluripotent stem cell (hPSC); Circulating tumor cells (CTCs); Patient Derived Organoid (PDO); Rhabdomyosarcomas (RMS); Tumor-infiltrating lymphocytes (TILs); Food and Drug Administration (FDA); β-hydroxybutyrate (BHB); Cancer-associated fibroblasts (CAFs).
